# Epidemiology and treatment of phalangeal fractures: conservative treatment is the predominant therapeutic concept

**DOI:** 10.1007/s00068-020-01397-y

**Published:** 2020-05-25

**Authors:** Laura Kremer, Johannes Frank, Thomas Lustenberger, Ingo Marzi, Anna Lena Sander

**Affiliations:** 1 Department of Trauma, Hand and Reconstructive Surgery, University Hospital Frankfurt, Frankfurt am Main, Germany; 2 Klinik für Unfall-, Hand- und Wiederherstellungschirurgie, Universitätsklinikum Frankfurt, Theodor-Stern-Kai 7, 60590 Frankfurt am Main, Germany

**Keywords:** Phalangeal fractures, Epidemiology, Fracture type, Classification, Treatment

## Abstract

**Purpose:**

Despite the high number of patients with phalangeal fractures, evidence-based recommendations for the treatment of specific phalangeal fractures could not be concluded from the literature. The purpose of the present study was to assess current epidemiological data, classification of the fracture type, and mode of treatment.

**Methods:**

This study presents a retrospective review of 261 patients with 283 phalangeal fractures ≥ 18 years of age who were treated in our level I trauma centre between 2017 and 2018. The data were obtained by the analysis of the institution’s database, and radiological examinations.

**Results:**

The average age of the patients was 40.4 years (range 18–98). The ratio of male to female patients was 2.7:1. The two most typical injury mechanisms were crush injuries (33%) and falls (23%). Most phalangeal fractures occurred in the distal phalanx (P3 43%). The 4th ray (D4 29%) was most frequently affected. The P3 tuft fractures, and the middle phalanx (P2) base fractures each accounted for 25% of fracture types. A total of 74% of fractures were treated conservatively, and 26% required surgery, with Kirschner wire(s) (37%) as the preferred surgical treatment. The decision for surgical treatment correlated with the degree of angular and/or rotational deformity, intraarticular step, and sub-/luxation of specific phalangeal fractures, but not with age and gender.

**Conclusions:**

Our findings demonstrated the popularity of conservative treatment of phalangeal fractures, while surgery was only required in properly selected cases. The correct definition of precise fracture pattern in addition to topography is essential to facilitate treatment decision-making.

## Introduction

Phalangeal fractures are common injuries and can be complicated by deformity without sufficient treatment and stiffness due to inadequate immobilisation or overtreatment [1–3]. Despite the high number of patients, evidence-based recommendations for the treatment of specific phalangeal fractures could not be concluded from the literature and randomised controlled trials are prevented from being performed due to the wide range of variation in fracture patterns and the associated variables that are thought to affect treatment and outcome [4]. In this context, it is also evident that there is a lack of a comprehensive and generally accepted classification [5–8]. This study was performed to evaluate current epidemiological data, classification of the fracture type, and mode of treatment.

## Patients and methods

Institutional review board approval (GN239/16) was obtained prior to initiating this retrospective study. The study included all patients ≥ 18 years of age with phalangeal fractures who were treated in our level I trauma centre over a 2-year period (2017–2018). An electronic ICD-10 search was conducted and 261 patients were identified. The data were collected by the analysis of the institution’s database, and radiological examinations. Information obtained included age, gender, injury mechanism, injured side, phalanx, ray, fracture type, and mode of treatment. Injury mechanism was divided into the following categories: crush injury, jam injury (axial loading to the tip of the finger), distorsion, hyperextension, fall from standing or seating height, violent assault, bicycle accident, motor vehicle accident, and other/unclear. All patients underwent standard of care imaging that included radiographs in two planes. Computed tomography (CT) imaging was used in 6% (17/283) of fractures, of which 53% (9/17) were intraarticular, and 47% (8/17) extraarticular. The phalangeal fractures were classified according to the topography using preoperative radiological imaging (Table 1) [3, 9]. Stable and reducible fractures, which do not displace in a cast in the first 5–14 days after reduction, were treated nonoperatively. The indication for surgical fixation included angular and/or rotational deformity, intraarticular impression and/or step > 2 mm, and sub-/luxation. All operative patients were treated by surgeons specialised in orthopaedic trauma care. Statistical evaluation was performed using Chi-square test. Values of *p* < 0.05 were considered statistically significant.

**Table 1:** Topographical classification of phalangeal fractures

P3
Tuft fracture
Shaft fracture
Base fracture
Volar avulsion (profundus avulsion)
Dorsal avulsion (mallet fracture)
Lateral avulsion
Base fracture
P1/P2
Condylar fracture
Unicondylar fracture
Bicondylar fracture
Neck fracture
Shaft fracture
Base fracture
P2 volar avulsion
P2 dorsal avulsion
P1/P2 lateral avulsion
P2 pilon fracture
P1 base fracture

## Results

The average age was 40.4 years (range 18–98) consisting of 90% (235/261) adult patients (< 65 years), and 10% (26/261) elderly patients (≥ 65 years). The ratio of male to female patients was 2.7:1. The average age of males was 39 years (range 18–83) with 94% (178/190) adult patients, and 6% (12/190) elderly patients, and 44.1 years (range 18–98) for females with 80% (57/71) adult patients, and 20% (14/71) elderly patients. In the elderly population, significantly more female compared to male patients were found (*p* = 0.001). The most typical injury mechanism was crush injury (33%, 87/261) followed by accidental fall (23%, 61/261), and jam injury (15%, 40/261). 29% (76/261) of the accidents occurred at work. The ratio of right (53%, 138/261) to left (47%, 123/261) hands was 1.1:1. The 261 patients had 283 fractures, including 7% (18/261) of patients with multiple fractures. Of these, 83% (15/18) had two fractures, 11% (2/18) three fractures, and 6% (1/18) four fractures, with the multiple fractures most commonly (72%, 13/18) at the same level [e.g., adjacent distal phalanx (P3)] (Table 2).

**Table 2:** Epidemiological and injury details

Number of patients	261
Age (years)	40.4 (18–98)
Gender (male:female)	2.7:1
Injury mechanism	
Crush injury	33% (87/261)
Fall from standing or seating height	23% (61/261)
Jam injury	15% (40/261)
Violent assault	6% (16/261)
Bicycle accident	6% (15/261)
Hyperextension	5% (14/261)
Other/unclear	5% (12/261)
Distorsion	4% (10/261)
Motor vehicle accident	2% (6/261)
Injured side	
Right	53% (138/261)
Left	47% (123/261)
Number of fractures	283
Patients with multiple fractures	7% (18/261)
2	83% (15/18)
3	11% (2/18)
4	6% (1/18)

**Fig. 1:**
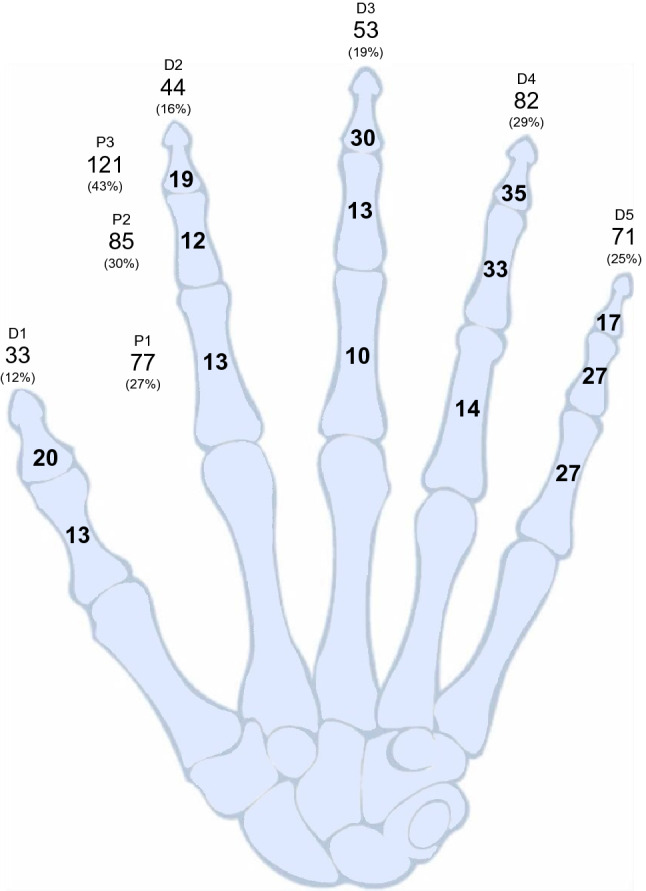
The distribution of phalangeal fractures by phalanx and ray

43% (121/283) of the phalangeal fractures occurred in the P3, 30% (85/283) in the middle phalanx (P2), and 27% (77/283) in the proximal phalanx (P1). The 4th ray (D4 29%, 82/283) was most frequently affected followed by the 5th (D5 25%, 71/283), and the 3rd (D3 19%, 53/283). Evaluating the distribution of phalanx versus ray, the P3 of the D4 (12%, 35/283) was most commonly injured followed by the P2 of the D4 (12%, 33/283), and the P3 of the D3 (11%, 30/283) (Fig. 1).

Analysing the distribution of phalanx versus fracture type, P3 tuft fractures (25%, 72/283), and P2 base fractures (25%, 70/283) were the most common fracture types followed by P1 base fractures (17%, 48/283) (Table 3). Subdividing the base, the most typical fracture type of the P1 was the base fracture (65%, 31/48) compared to volar avulsion (80%, 56/70) in the P2, and base fracture (44%, 17/39) in the P3 (Table 4).

**Table 3:** Distribution of phalanx versus fracture type

P3	
Tuft fracture	25% (72/283)
Shaft fracture	4% (10/283)
Base fracture	14% (39/283)
P2	
Condylar fracture	1% (2/283)
Neck fracture	1% (3/283)
Shaft fracture	4% (10/283)
Base fracture	25% (70/283)
P1	
Condylar fracture	1% (4/283)
Neck fracture	0.4% (1/283)
Shaft fracture	8% (24/283)
Base fracture	17% (48/283)

**Table 4:** Distribution of base versus fracture type

P3 base	
Base fracture	44% (17/39)
Dorsal avulsion	31% (12/39)
Volar avulsion	21% (8/39)
Lateral avulsion	5% (2/39)
P2 base	
Volar avulsion	80% (56/70)
Pilon fracture	9% (6/70)
Lateral avulsion	9% (6/70)
Dorsal avulsion	3% (2/70)
P1 base	
Base fracture	65% (31/48)
Lateral avulsion	33% (16/48)
Volar avulsion	2% (1/48)

41% (115/283) of fractures were intraarticular, and 59% (168/283) extraarticular, with 4% (12/283) of fractures resulting in joint luxation. Of these, 75% (9/12) of fractures occurred in the P2, and 25% (3/12) in the P3, with volar avulsion (92%, 11/12) as most typical fracture type. Closed fractures accounted for 87% (247/283) of fractures compared to 13% (36/283) open fractures with significant soft tissue injuries communicating with the fractures.

74% (210/283) of the fractures could be treated conservatively, respectively 26% (73/283) with surgery. Reviewing the relationship between age and mode of treatment, 75% (188/251) of the adult patients were managed without surgery, and 25% (63/251) with surgery compared to 69% (22/32), and 31% (10/32) of the elderly patients. Evaluating the distribution of gender versus mode of treatment, males were treated conservatively in 74% (150/204), and with surgery in 26% (54/204) versus 76% (60/79), and 24% (19/79) for females. The differences were not statistically significant. Therefore, in the present study population, the decision for surgical treatment was independent of age (*p* = 0.454) and gender (*p* = 0.676).

Analysing the distribution of phalanx versus mode of treatment, 65% (50/77) of P1 fractures could be treated conservatively, and 35% (27/77) with surgery compared to P2 fractures with 87% (74/85) versus 13% (11/85), and P3 fractures with 71% (86/121) versus 29% (35/121) (Table 5).

**Table 5:** Distribution of phalanx versus mode of treatment

	Operative	Nonoperative
P3	29% (35/121)	71% (86/121)
P2	13% (11/85)	87% (74/85)
P1	35% (27/77)	65% (50/77)

Kirschner wire(s) (37%, 27/73) predominated the surgical treatment followed by sutures of the nail bed (22%, 16/73) and locking plates (16%, 12/73) (Table 6).

**Table 6:** Mode of surgical treatment

Kirschner wire(s)	37% (27/73)
Suture of the nail bed	22% (16/73)
Locking plate	16% (12/73)
Suture anchor	8% (6/73)
Dynamic distraction external fixator	8% (6/73)
Screw(s)	7% (5/73)
Mini external fixator	1% (1/73)

## Discussion

Age and gender are both important factors for defining the risk of sustaining phalangeal fractures [10]. Young males and elderly females are known to be most susceptible for this injury [2, 10, 11]. Males remain at a relatively greater risk for phalangeal fractures than females up until the age of 60 likely resulting from increased behavioural risk factors such as participation in higher risk sport and occupational activities until the age of retirement [10, 12]. Females begin to show a greater risk after the age of 65 due to a longer life expectancy with exponential increase in the incidence of falls and osteoporosis [13, 14]. The age and gender distribution of the present study was congruent with those published demonstrating predominantly adult male patients and a larger female proportion in the elderly population.

The two most typical injury mechanisms are direct blow and accidental fall, with a variable distribution in the different age groups [4, 15]. De Jonge et al. showed that falls are responsible for most of phalangeal fractures in patients over 70 years, in contrast to another study, in which the majority of the fractures were caused by a direct, or crush injury, even in the retired population [1, 15]. Our data demonstrated that adult patients sustained predominantly crush injury whereas falls occurring primarily in elderly patients consistent with the study of de Jonge et al. [1].

The ratio of right and left hands of phalangeal fractures has been reported to be 1:1 in previous studies which is similar to the present study population [14, 16]. Hence, right-hand dominance does not result in higher incidence of phalangeal fractures of the right hand [14, 17].

The D5, as border ray, has been shown to be most frequently affected [4, 15]. However, we noticed that our ray profile differed, in which the D4 was most commonly injured confirming the theory that the incidence of phalangeal fractures is proportional to the length of the digit [18]. Congruently, most phalangeal fractures occurred in the P3 reflecting the kind of injury mechanism (crush injury) in our study population.

Few data are available in the literature regarding fracture type of phalangeal fractures [15]. Stanton et al. found that the P3 tuft and base were common sites of injury, in which intraarticular fractures were rare [15]. Our study revealed a different distribution of fracture types which were also classified according to the topography (Table 1) [3, 9]. The two most frequent fracture types were P3 tuft and P2 base fractures. The most vulnerable parts of the P1 and P2 were their bases accounting for 73% of fractures. Subdividing the base, the most common fracture type of the P1 was the base fracture compared to volar avulsion in the P2. Intraarticular fractures represented a large proportion of all fractures, which could be explained, in part, by the different injury mechanism.

The majority of phalangeal fractures can be treated without an operation [2, 15, 19]. Our data were consistent with the conservative trend, but the percentage of fractures treated surgically was higher compared to the current literature. This could be due to the higher number of intraarticular fractures in our trauma centre, which accounted for 34% of fractures requiring surgery.

Age has been shown to be the most important variable in determining whether operative or nonoperative management is appropriate, with advanced age more predictive of nonoperative management [20]. Contrarily, in the present study, elderly patients were just as likely to get surgical treatment as adult patients due to a variety of reasons. First, the high percentage of intraarticular fractures (34%). Second, it was also due to a personal request of the elderly patients who are now more active than ever and often prefer surgical treatments that do not hamper their activities.

Kirschner wires and screw-plate fixation generally predominate in the various modes of surgical treatment [4, 15, 21]. Similarly, in our study, Kirschner wire(s) were required in most cases, while the proportion of locking plates was lower. The further fixation methods included suture anchor, dynamic distraction external fixator, screw(s), and mini external fixator in that order reflecting the kind of fracture pattern with a high number of intraarticular fractures in our study population.

Some limitations must be considered for the present study. First, the study design was retrospective. Second, our data provided no information on outcomes. Even though this study contributes to currently available epidemiological data, the definite answer regarding appropriate algorithm for phalangeal fractures requires prospective long-term outcome studies.

## Conclusion

In conclusion, our results validated the trend of conservative treatment for the vast majority of phalangeal fractures. Surgery, however, was required in properly selected cases depending on the degree of angular and/or rotational deformity, intraarticular impression and/or step, and sub-/luxation, with the use of Kirschner wire(s) as preferred surgical treatment. The correct definition of precise fracture pattern in addition to topography is essential to facilitate clinical treatment decision-making.
